# A Comparative Study of Prism Adaptation Using Virtual Reality and Conventional Prism Glasses

**DOI:** 10.7759/cureus.91378

**Published:** 2025-09-01

**Authors:** Kimika Anan, Akira Nakashima, Takefumi Moriuchi, Makoto Fujimura, Toshio Higashi

**Affiliations:** 1 Department of Health Sciences, Graduate School of Biomedical Sciences, Nagasaki University, Nagasaki, JPN; 2 Department of Occupational Therapy Science, Graduate School of Biomedical Sciences, Nagasaki University, Nagasaki, JPN; 3 Department of Integrated Science and Technology, Graduate School of Integrated Science and Technology, Nagasaki University, Nagasaki, JPN

**Keywords:** aftereffects assessment, healthy adults, prism adaptation therapy, unilateral spatial neglect, virtual reality rehabilitation

## Abstract

Background: Unilateral spatial neglect (USN) is a symptom of cerebrovascular disorders characterized by impaired attention to the space opposite a cerebral hemisphere lesion. Prism adaptation (PA) is a rehabilitation technique considered effective for treating USN. PA involves performing tasks while wearing prism glasses, which shift the visual field and help correct spatial perception. However, methods tailored to the severity of USN have not been fully established. Virtual reality (VR) has been proposed as a potential solution. This study aimed to compare the effects of a VR-based PA system and conventional prism glasses under equivalent task conditions.

Methods: The participants included 40 healthy, right-handed adults. Each participant completed three conditions: control, prism glasses, and VR. The order of these conditions was counterbalanced across participants. A one-week washout period was implemented between conditions. For evaluation, the open-loop pointing (OLP) test, the landmark task, and the line bisection task were administered as baseline assessments. Participants then performed the PA task. After the PA task, the same evaluations were repeated as post assessments. A leftward deviation following the PA was considered indicative of successful adaptation. A chi-square test was used to analyze changes in OLP test scores between the pre- and post assessments. Furthermore, for participants with successful PA, a one-way analysis of variance (ANOVA) was conducted to compare the magnitude of aftereffects across conditions.

Results: Among the 40 participants, 19 showed a leftward deviation in the prism glasses condition and 34 in the VR condition, indicating a statistically significant difference. Among the 17 participants with successful PA in both the prism and VR conditions, a one-way ANOVA revealed a significant main effect in OLP test score changes. Post hoc analysis revealed significant differences between the control condition and both the prism glasses and VR conditions.

Conclusion: The VR-based PA system induced PA in more participants than conventional prism glasses and produced comparable aftereffects. These findings support the utility of VR-based PA and suggest its potential to advance future rehabilitation methods.

## Introduction

Unilateral spatial neglect (USN) is commonly observed in patients with cerebrovascular disorders and is characterized by difficulty attending to the space contralateral to a cerebral hemisphere lesion. It is defined as the inability to report, respond to, or orient toward stimuli presented on the contralesional side [1,2]. USN occurs in approximately 48% of patients with stroke, with a particularly high prevalence of left-sided USN following right hemisphere damage [3]. A systematic review examining the relationship between USN and physical recovery reported that its presence is associated with poorer improvement in upper limb function [4]. Furthermore, greater severity of USN has been shown to negatively impact the recovery of activities of daily living (ADL) [5]. Given its significant impact on post-stroke motor recovery and daily functioning, accurate evaluation and effective treatment of USN are critical in neurorehabilitation.

Among rehabilitation interventions for USN, prism adaptation (PA) has attracted considerable attention. PA involves performing tasks while wearing prism glasses, which induce a visual field deviation and subsequently cause a shift in the subjective midline after removal [6,7]. When applied to patients with USN, this technique is referred to as PA therapy. Previous studies have reported that PA therapy produces immediate effects in patients with chronic-stage USN during tabletop assessments and maintains those effects for several hours post-intervention [8,9]. Additionally, a randomized controlled trial (RCT) involving patients with subacute USN demonstrated that two weeks of PA therapy significantly improved USN symptoms and contributed to better ADL outcomes [10].

However, current PA therapy using prism glasses presents several limitations. First, the degree of deviation depends on the lens used, making it difficult to adjust the settings precisely based on USN severity. As a result, the angle of deviation varies across studies [11-13]. Moreover, the adaptation process and its aftereffects cannot be quantitatively measured, making it challenging to track the progress of USN improvement objectively during the intervention. Additionally, the number of reaching movements used in PA interventions lacks consistency [6,8,14]. Therefore, current prism-glasses-based methods are not sufficiently established for tailored applications based on USN severity, highlighting the need for more adaptable approaches.

One promising alternative is the use of virtual reality (VR). Ramos et al. employed two different VR-based PA methods and compared their aftereffects with those of conventional prism glasses [15]. Their results indicated that VR-based PA produced greater aftereffects than the conventional method. Although other studies have used VR-based PA, few have directly compared it with conventional prism glasses under equivalent task conditions. Consequently, while previous studies, including Ramos et al. [15], reported promising outcomes for VR-based PA, its generalizability, long-term clinical relevance, and reliability across diverse assessment tasks have yet to be thoroughly examined.

Based on these findings, this study aims to compare the effects of PA using VR and conventional prism glasses under identical task conditions, with the goal of informing the methodological development of PA therapy.

## Materials and methods

Participants

The study comprised 40 participants who were healthy, right-handed adults (26 females, 14 males; mean age: 23.0 ± 3.5 years), all with normal or corrected-to-normal vision and no neurological symptoms. Prior to the experiment, all participants received a detailed explanation of the study's purpose and procedures and provided written informed consent. This study was approved by the Ethics Committee of the Graduate School of Biomedical Sciences, Nagasaki University, Nagasaki, Japan (approval number 19080802). Each participant completed three experimental conditions: control, prism lens (PL), and VR.

Prism adaptation

Following previous studies, the prism deviation angle was set to 20 diopters (approximately 11.3°) to the right, a commonly used setting for inducing PA [6,12,16]. In this study, both the prism glasses and VR systems applied a 20-diopter rightward deviation to induce PA.

Experimental task

PL Condition

**Figure 1:**
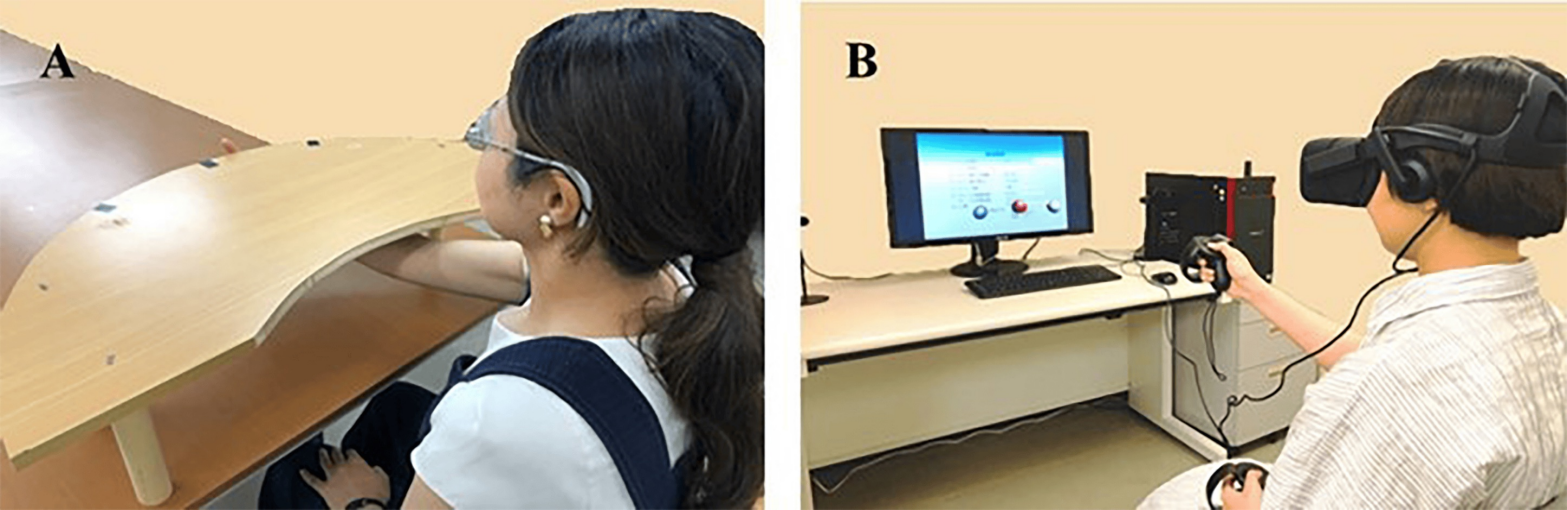
Prism adaptation task (A) Prism lens condition; (B) Virtual reality-based prism adaptation (VRPA) condition This figure has been created by the authors.

In the PL condition, participants were seated in front of a table with three targets arranged in an arc. While wearing prism glasses, participants were instructed to perform 96 reaching movements. Auditory cues from a metronome were presented every three seconds to signal the timing for each reach. Participants began with their right hand positioned in front of the sternum and were instructed to reach sequentially with their right index finger from below the table toward the targets (Figure 1A).

The prism glasses used in this condition were fitted with 20-diopter Fresnel membrane prisms (base-left) (Fresnel Prism and Lens Co., Bloomington, MI, USA) attached to the lenses of both eyes. 

VR Condition

In the VR condition, the head-mounted display (HMD) was the Oculus Rift (Oculus VR, Inc., Meta Platforms, Inc., Menlo Park, CA, USA), and hand movements were tracked using Oculus Touch controllers (Oculus VR, Inc.). The system was programmed using Unity (Unity Technologies, San Francisco, CA, USA).

This immersive VR system simulated the same repeated reaching task as in the PL condition but within a virtual space (Figure 1B). Notably, the VR system allowed fine adjustment of the deviation angle in 1-diopter increments via PC settings, along with customization of target distance, height, number, appearance timing, and number of sets. It also enabled storage of settings and performance data, as well as recording and analysis of the error between the target and the participant's reach, facilitating observation of the adaptation process. Throughout the experiment, participants were instructed to keep their trunk and head facing forward. A chair with back support was used to minimize trunk rotation, and the HMD fit was adjusted to align with each participant’s head center to reduce misalignment during the VR task.

**Figure 2:**
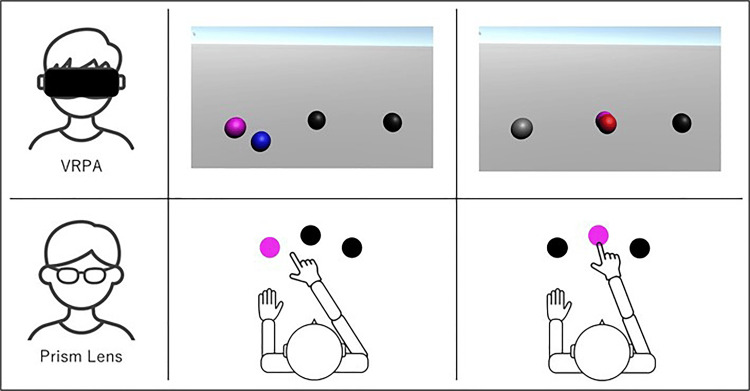
Comparison of task performance before and after prism adaptation (PA) between the virtual reality-based prism adaptation (VRPA) task and prism lens task This figure illustrates the changes in performance from pre- to post-PA for two types of tasks: a virtual reality (VR)-based task and a conventional (non-VR) task. The comparison highlights the effect of PA on task outcomes in both modalities. Data were collected before and after the PA phase to evaluate the transfer or generalization effects induced by each task type. This figure has been created by the authors.

In the VR condition, three gray spherical targets were displayed in an arc directly in front of the participant in the VR space. The reaching cue was presented visually by changing the target color to purple every three seconds. Participants, under the prism-deviated condition, performed 96 reaching movements toward the purple targets. They were instructed to press a button on the controller at the location where they believed the target was located. If the reached point deviated from the target, it appeared in blue (Figure 2A); if it overlapped exactly, it appeared in red (Figure 2B), providing immediate visual feedback.

In both conditions, participants received immediate visual feedback after each pointing movement. In the PL condition, feedback was provided by viewing the position of the index finger relative to the target through the PLs. In the VR condition, feedback was provided by the appearance of a virtual sphere at the location corresponding to the participant’s finger position. Although the presentation differed, both conditions offered comparable feedback on motor accuracy.

Control Condition

In the control condition, participants performed the same task as in the PL condition but without any prism deviation. Participants were seated in a chair and instructed to remain relaxed and keep their heads as still as possible throughout the experiment. All reaching movements were performed using the right hand.

Assessment of Aftereffects

Following previous studies [17-20], we used three assessments to evaluate PA and its aftereffects: the open-loop pointing (OLP) test, the landmark task, and the line bisection task.

OLP test

This test measures deviation in the perceived subjective midline. Participants were instructed to point, with their eyes closed, to what they perceived as the center of a whiteboard placed parallel to the frontal plane. The actual center of the board was defined as 0, and the deviation was measured in 0.1 cm increments. Values to the right were recorded as positive, and those to the left as negative.

Landmark task

Participants verbally indicated whether the center dividing line of a horizontal line appeared to shift to the right or left. Each sheet included 16 lines (20 cm in length), 10 of which were precisely bisected, while the remaining six were offset by 1, 3, or 5 mm to the right or left. Unaware of the offset, participants judged the perceived direction of the center. The percentage of "right-shifted" responses among the 10 truly centered lines was used for analysis.

Line bisection task

Participants were instructed to mark the midpoint of each of 10 randomly arranged 20-cm horizontal lines on a sheet. The distance from the actual center to the marked point was measured in 0.1 cm increments. Positive values indicated rightward deviations, and negative values indicated leftward deviations. The mean deviation of all 10 lines was used as the final score.

Three post-adaptation assessments were selected to capture different components of PA: the OLP test, the landmark task, and the line bisection task. The OLP test primarily measures immediate sensorimotor readjustment, the landmark task assesses perceptual midline judgments, and the line bisection task evaluates spatial attention bias. The assessment order was fixed, with all participants completing the OLP test, landmark task, and line bisection task in that sequence. Immediate visual feedback was provided in both conditions. In the PL condition, participants visually confirmed the position of their index finger relative to the target, while in the VR condition, a virtual sphere appeared at the corresponding finger position. Although the presentation modes differed, both feedback formats conveyed equivalent information regarding motor accuracy. Given its high sensitivity for detecting short-term sensorimotor aftereffects, particularly in healthy participants, the OLP test was used as the primary criterion for defining successful PA in this study.

Experimental protocol

Each participant completed all three conditions (control, PL, and VR), with the order of conditions counterbalanced. A washout period of one week was established between conditions.

**Figure 3:**
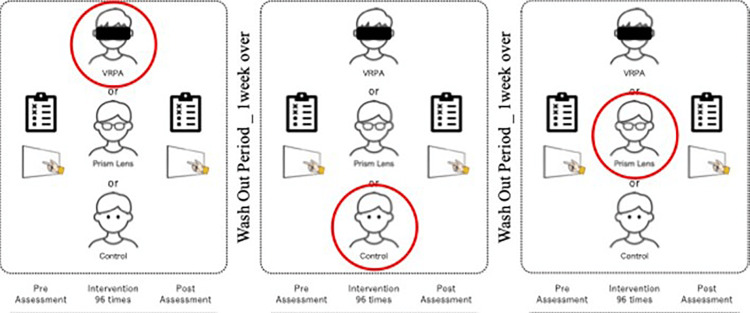
Experimental protocol Each participant completed all three experimental conditions (VRPA task, prism lens task, and control task). The order of task execution was counterbalanced across participants to minimize order effects. In each session, a pre-assessment was conducted prior to intervention, followed by the designated task, and then a post-assessment was conducted immediately. Each experimental session was separated by a one-week washout period to avoid carryover effects. The figure illustrates one example sequence of the experimental schedule. VRPA: virtual reality-based prism adaptation This figure has been created by the authors.

In each session, the OLP test, the landmark task, and the line bisection task were administered first as pre-tests. After completing the experimental task, participants waited with their eyes closed for approximately 10 seconds while the post-test environment was prepared. The same three assessments were subsequently repeated as post tests (Figure 3).

Statistical analysis

We used the OLP test as the primary indicator of whether PA occurred. Participants who demonstrated a leftward deviation after PA in either the PL or VR condition were considered to have successfully adapted. Based on the change in the OLP test from pre- to post test, participants were classified into “with leftward deviation” and “without leftward deviation” groups, and chi-square tests were performed.

For participants who demonstrated successful adaptation, the degree of aftereffects between conditions was compared using change scores (post-pre) from the OLP test, landmark task, and line bisection task. A one-way analysis of variance (ANOVA) with “condition” as a main factor was conducted. Bonferroni correction was applied for post-hoc analysis. Statistical analyses were conducted using IBM SPSS Statistics software, version 22.0 (IBM Corp., Armonk, NY, USA), with the significance level set at p < 0.05.

## Results

Comparison of PA based on the change in the OLP test

Among the 40 participants, 19 (47.5%) exhibited a leftward deviation in the OLP test under the PL condition, whereas 34 (85.0%) exhibited a leftward deviation under the VR condition. A chi-square test was conducted to examine the association between the condition and the presence of leftward deviation, revealing a significant difference between the PL and VR conditions (p = 0.000).

Comparison of aftereffects based on changes in each assessment

**Figure 4:**
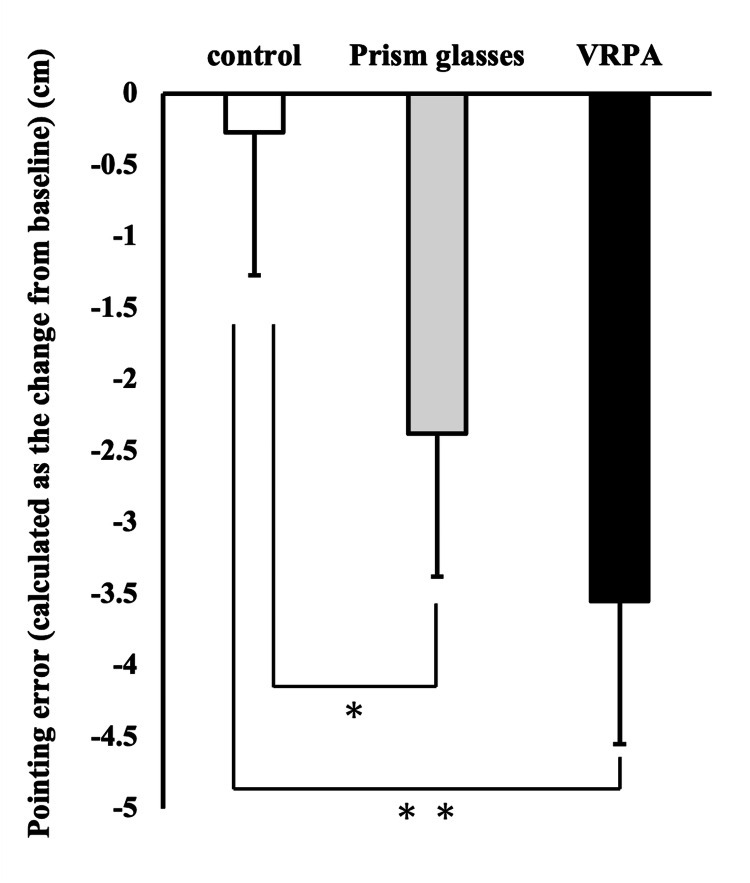
Comparison of after effects across task conditions The graph illustrates the magnitude of aftereffects observed under each task condition, calculated as the change from baseline. * p<0.05; **p<0.01 VRPA: virtual reality-based prism assessment

A total of 17 participants (42.5%) were identified as having successfully adapted in both the PL and VR conditions. A one-way ANOVA was conducted using data from these 17 participants. A significant main effect was observed for the change in the OLP test (F(2, 32) = 12.365, p = 0.000). Post-hoc analysis revealed significant differences between the control and PL conditions (p = 0.027), as well as between the control and VR conditions (p = 0.001) (Figure 4). No significant main effects were observed in either the landmark task or the line bisection task.

## Discussion

This study aimed to compare the effectiveness of a previously developed VR-based prism PA system with that of a conventional PL under identical PA task conditions. The results demonstrated that a greater proportion of participants exhibited leftward deviation in the OLP test under the VR condition than under the PL condition. Furthermore, among participants who exhibited leftward deviation in both conditions, a similar degree of deviation in the subjective midline was observed in both experimental conditions relative to the control. These findings suggest that the VR system may induce PA more effectively than conventional PLs and may produce aftereffects of a similar magnitude.

One possible reason for the greater effectiveness of the VR system in inducing PA is the immersive nature of the HMD used in the study. Immersive VR environments can block external distractions and enhance attentional focus, potentially increasing task engagement during PA. Previous studies have reported that immersive VR enhances attentional capacity, which may, in turn, facilitate PA [21-23]. Moreover, awareness of the visual shift has been shown to influence the success of PA; specifically, PA is less likely to occur when the shift is noticed and more likely when it is not [21,24]. Thus, the immersive nature of VR may have reduced the perceived discomfort associated with visual displacement, making adaptation easier than with conventional PLs.

Previous research involving healthy individuals has also shown that immersive VR environments can produce performance outcomes comparable to real-world tasks, supporting the potential of VR for evaluating and rehabilitating USN [25-28].

With regard to aftereffects, both the VR and PLs conditions produced leftward deviations in the OLP test, consistent with previous findings using conventional PA methods [18,25,29]. The OLP test is widely used as a standard measure to assess PA-induced changes. PA is known to cause a shift between somatosensory and visual perception, resulting in a leftward deviation of the subjective midline. The current results are consistent with these findings, indicating that VR can generate aftereffects comparable to those induced by conventional PLs.

In contrast, no significant main effects were found in either the landmark or line bisection tasks. Previous studies have shown that patients with USN are more susceptible to PA-induced changes and that PA can reduce visual neglect symptoms [6]. However, it has also been reported that once PA is achieved, de-adaptation can occur rapidly after the removal of the prism [22], and that spatial cognition is largely vision-dependent. Therefore, the limited persistence of aftereffects in healthy participants, unlike in patients with USN, may account for the lack of significant changes in these two tasks.

The three post-adaptation tasks employed in this study were designed to evaluate complementary aspects of PA: the OLP test assessed immediate sensorimotor realignment, the landmark task measured perceptual midline judgment, and the line bisection task evaluated spatial attentional bias. Among these, significant changes were observed only in the OLP test. This finding is consistent with previous research indicating that the OLP test is particularly sensitive to short-term sensorimotor aftereffects in healthy individuals [30]. One possible explanation is that the OLP test directly reflects recalibration of the visuomotor mapping formed during adaptation. By contrast, the landmark and line bisection tasks involve higher-order perceptual and attentional processes, which may show greater intra-individual variability and may be less susceptible to short-term adaptation effects. Furthermore, the extent to which sensorimotor adaptation transfers to perceptual or attentional domains may depend on adaptation strength, task complexity, and participant characteristics. These findings underscore the importance of employing multiple assessment measures to capture the multifaceted nature of PA, and they suggest that, in healthy individuals, immediate post-adaptation changes are more readily detected at the sensorimotor level.

All participants were assessed in a fixed order, which ensured procedural consistency but may have introduced an order effect. Performing certain tasks earlier may have influenced performance or the manifestation of adaptation in subsequent tasks. Future studies should consider randomizing or counterbalancing task order to minimize this potential bias.

This study partially replicates the findings of Ramos et al. [15], who reported stronger aftereffects in VR-based PA compared to conventional prism exposure. Both studies demonstrated superior outcomes in the VR condition, supporting the notion that immersive VR can enhance PA. However, the manifestation of this enhancement differed between the two studies: Ramos et al. primarily observed larger aftereffect magnitudes, whereas the present study found a higher proportion of participants achieving adaptation, with similar deviation magnitudes among those who adapted in both conditions. Such differences may reflect variations in methodological parameters, including the deviation angle, trial number, feedback modality, and the operational definition of “successful” adaptation. Integrating the findings from both studies suggests that VR-based PA may be particularly effective at increasing the likelihood of adaptation, while the magnitude of aftereffects may depend more on individual sensitivity and specific task parameters. Future refinements, such as optimizing feedback and exposure conditions, may further improve both adaptation rates and aftereffect magnitudes in VR-based PA.

Nevertheless, several limitations of the present study should be acknowledged. First, the persistence of aftereffects over time was not examined. Second, the findings were derived exclusively from healthy adults, thereby limiting their generalizability to clinical populations such as patients with USN. Although previous studies have suggested the effectiveness of VR-based PA in such patients [31], future research should address these limitations by investigating aftereffects under varying trial numbers and deviation angles and by optimizing experimental parameters accordingly. Moreover, the VR system’s capacity to record detailed data on the PA process should be utilized to quantitatively evaluate adaptation and aftereffects. Finally, additional clinical studies involving patients with USN are warranted to establish the clinical utility of VR-based PA systems.

## Conclusions

This study demonstrated that a VR-based PA system using an HMD was more effective in inducing adaptation than a conventional PL setup under identical task conditions. Notably, a higher proportion of participants exhibited a leftward deviation in the subjective midline under the VR condition, and both VR and PL conditions produced comparable aftereffects as measured by the OLP test. These results, although obtained from healthy adults, suggest that immersive VR may enhance attentional engagement and alleviate discomfort associated with visual displacement, thereby facilitating more robust adaptation. Although no significant effects were found in the landmark and line bisection tasks, these outcomes may reflect the transient nature of aftereffects in healthy individuals, highlighting the need for further investigation in clinical populations such as patients with USN. The immersive VR system's ability to replicate real-world sensorimotor interactions and capture detailed behavioral data offers promising avenues for both assessment and rehabilitation. In conclusion, the findings underscore the potential of VR-based PA systems as a viable and effective tool for inducing PA and generating measurable aftereffects. Future studies should explore their applicability in clinical settings, optimize parameters to enhance efficacy, and assess their long-term benefits in patient populations. The integration of immersive VR technology into neurorehabilitation may represent a meaningful advancement in the treatment of spatial cognition disorders.
